# Supplementary material for: Identification of photoperiod-induced specific miRNAs in the adrenal glands of Sunite sheep (Ovis aries)
Source: Front Vet Sci. 2022 Jul 22;9:888207. doi: 10.3389/fvets.2022.888207 (PMC9354845; doi:10.3389/fvets.2022.888207)
Supplement: Supplementary file 1 [file Data_Sheet_1.ZIP › Supplementary file/miRDeep2 file.zip!/miRDeep2 file/Know_Structure/SP42A2/oar-mir-23b.pdf]

miRBase precursor : oar-mir-23b
Total read count : 24171
oar-miR-23b read count : 24132
remaining reads : 39

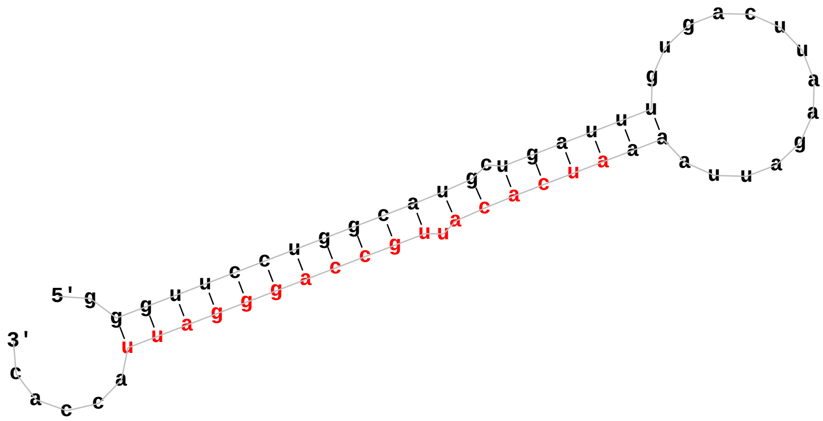

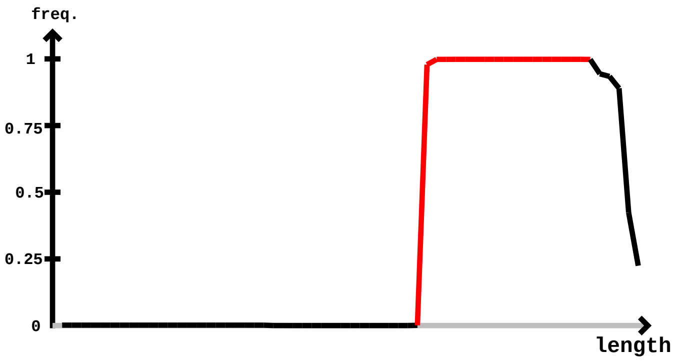


```
                                     oar-miR-23b
5'- ggguuccuggcaugcugauuugugacuuaagauuaaaaucacauugccagggauuaccac   -3'    exp
    ((((((((((((((.((((((..............)))))))).))))))))))..))..    reads      mm         sample
    ggguuccuggcaugcugau.........................................    1          0          seq
    ggguuccuggcaugcugauu........................................    5          0          seq
    ggguuccuggcaugcugauuu.......................................    30         0          seq
    ggguuccuggcaugcugauuugu.....................................    2          0          seq
    ....................ugugacuuaagauuaaaau.....................    1          0          seq
    ....................................aaucacauugccagggauu.....    6          0          seq
    ....................................aaucacauugccagggauuac...    3          0          seq
    ....................................aaucacauugccagggauuacc..    7          0          seq
    ....................................aaucacauugccagggauuacca.    1          0          seq
    ....................................aaucacauugccagggauuaccac    4          0          seq
    .....................................aucacauugccagggGu......    1          1          seq
    .....................................aucacauugccagggaC......    1          1          seq
    .....................................aucacauugccagggau......    9          0          seq
    .....................................aucacauugcUagggauu.....    2          1          seq
    .....................................aucacauugccagggauu.....    1292       0          seq
    .....................................aucacauugccUgggauu.....    1          1          seq
    .....................................aucacauugcUagggauua....    1          1          seq
    .....................................aucacauugccagggauua....    228        0          seq
    .....................................aucacauugccagggauuac...    1053       0          seq
    .....................................aucacauugccagggauuacc..    11099      0          seq
    .....................................aucacauugccagggauuacca.    4705       0          seq
    .....................................aucacauugccagggauuaccac    5236       0          seq
    ......................................ucacauugccagggauu.....    6          0          seq
    ......................................ucacauugccagggauua....    2          0          seq
    ......................................ucacauugccagggauuac...    18         0          seq
    ......................................ucacauugccagggauuacc..    159        0          seq
    ......................................ucacauugccagggauuacca.    107        0          seq
    ......................................ucacauugccagggauuaccac    183        0          seq
    .......................................cacauugccagggauuac...    1          0          seq
    .......................................cacauugccagggauuacc..    1          0          seq
    .......................................cacauugccagggauuacca.    1          0          seq
    ........................................acauugccagggauuacc..    5          0          seq
```
